# Volumetric bone mineral density (vBMD), bone structure, and structural geometry among rural South Indian, US Caucasian, and Afro-Caribbean older men

**DOI:** 10.1007/s11657-018-0473-1

**Published:** 2018-05-22

**Authors:** Guru Rajesh Jammy, Robert M. Boudreau, Tushar Singh, Pawan Kumar Sharma, Kristine Ensrud, Joseph M. Zmuda, P S Reddy, Anne B. Newman, Jane A Cauley

**Affiliations:** 1 Department of Epidemiology, Graduate School of Public Health, University of Pittsburgh, Pittsburgh, PA 15260 USA; 2 SHARE INDIA - MediCiti Institute of Medical Sciences, Ghanpur Village, Medchal District, Telangana India; 3 Division of Epidemiology and Community Health, University of Minnesota, Minneapolis, MN 55455 USA; 4 Department of Medicine, University of Minnesota, Minneapolis, MN 55455 USA; 5 Center for Chronic Disease Outcomes Research, VA Health Care System, Minneapolis, MN 55455 USA

**Keywords:** Race/ethnicity, Volumetric BMD, pQCT, Men, Osteoporosis, Epidemiology, India

## Abstract

***Summary*:**

Peripheral quantitative computed tomography (pQCT) provides biomechanical estimates of bone strength. Rural South Indian men have reduced biomechanical indices of bone strength compared to US Caucasian and Afro-Caribbean men. This suggests an underlying higher risk of osteoporotic fractures and greater future fracture burden among the rural South Indian men.

**Introduction:**

Geographical and racial comparisons of bone mineral density (BMD) have largely focused on DXA measures of areal BMD. In contrast, peripheral quantitative computed tomography (pQCT) measures volumetric BMD (vBMD), bone structural geometry and provides estimates of biomechanical strength. To further understand potential geographical and racial differences in skeletal health, we compared pQCT measures among US Caucasian, Afro-Caribbean, and rural South Indian men.

**Methods:**

We studied men aged ≥ 60 years enrolled in the Mobility and Independent Living among Elders Study (MILES) in rural south India (*N* = 245), Osteoporotic Fractures in Men Study (MrOS) in the US (*N* = 1148), and the Tobago Bone Health Study (*N* = 828).

**Results:**

The BMI (kg/m^2^) of rural South Indian men (21.6) was significantly lower compared to the US Caucasians (28) and Afro-Caribbean men (26.9). Adjusting for age, height, body weight, and grip strength; rural South Indian men compared to US Caucasians had significantly lower trabecular vBMD [− 1.3 to − 1.5 standard deviation (SD)], cortical thickness [− 0.8 to − 1.2 SD]; significantly higher endosteal circumference [0.5 to 0.8 SD]; but similar cortical vBMD. Afro-Caribbean men compared to US Caucasians had similar trabecular vBMD but significantly higher cortical vBMD [0.9 to 1.2 SD], SSIp [0.2 to 1.4 SD], and tibial endosteal circumference [1 SD],

**Conclusions:**

In comparison to US Caucasians, rural South Indian men have reduced bone strength (lower trabecular vBMD) and Afro-Caribbean men have greater bone strength (higher cortical vBMD). These results suggest an underlying higher risk of osteoporotic fractures and greater future fracture burden among rural South Indian men.

## Introduction

Global demographic patterns are changing with accelerated population aging fueled by declining fertility and increased longevity [1]. As per 2010 estimates, 8% of the world’s population (524 million) was 65 years and older and is expected to triple by 2050 [1]. Even though the developed countries have the oldest populations, the majority and rapidly aging populations are from less developed countries [1]. During 2010, about 65% of those aged 60 years and older lived in less developed countries, this is projected to increase to 80% by 2050 [2]. This demographic transition has important social, economic, and public health implications [3]. Even with increasing attention to the aging population throughout the globe, research related to aging has been primarily conducted in developed countries [4].

India is the second most populous country in the world and its older population segment is increasing dramatically. For example, approximately 8% of India’s population was aged 60 years and older in 2010 (93 million) and this population segment is projected to increase to 19% by 2050 (323 million) [5]. Of importance, more than two thirds of India’s elders live in rural areas [5]. The USA population aged 65 years and older between 2012 and 2050 is experiencing growth and will almost double [6]. The 18% of the 2010 population of the US aged 60 and over is estimated to increase to 27% by 2050 [7]. Trinidad and Tobago is also experiencing rapid aging. The 60 and over population in 2010 was 11% and is estimated to increase up to 32% by 2050 [7]. Trinidad and Tobago is among the top ten countries with the largest percentage increases in the share of the 60 and over population [7].

These increases in the older populations around the world will lead to large increases in the prevalence of many chronic conditions and degenerative diseases including osteoporosis [1]. A significant consequence of osteoporosis is fracture, which occurs due to imbalance of bone strength and force on the bone [8]. Osteoporosis has major and continued impact on the morbidity, quality of life, and mortality [9]. The International Osteoporosis Foundation estimates that one third of women and one fifth of men aged 50 years and over experience osteoporotic fractures [10].

Hip fractures increase with age and age standardized rates for men vary > 140-fold [11] across the world’s population. The factors contributing to this geographic variability in hip fracture rates are unknown, but may reflect differences in bone strength. A few studies have compared areal bone mineral density (aBMD) among different race and ethnic populations. For example, African-American and Afro-Caribbean men have higher aBMD at the hip and lumbar spine compared to US Caucasian and Hispanic men [12–15]. US Asians, Hong Kong Chinese and South Koreans have lower aBMD at lumbar spine compared to US Caucasian [15]. aBMD of Indian women aged 20–60 years, when compared with the US NHANES III data was about 27% lower [16]. However, aBMD is a two dimensional imaging technique that integrates cortical and trabecular BMD. Most of the evidence of geographical/racial comparisons has been limited to aBMD.

Peripheral quantitative computed tomography (pQCT) is an alternative technology developed for quantitative determination of bone density, structure, and structural geometry. To further understand potential geographical and racial differences, we compared pQCT measures of volumetric BMD (vBMD), bone structure, and structural geometry which are the indices of biomechanical strength among older men from three distinct race/ethnic populations: rural South Indian, US Caucasians, and Afro-Caribbean.

## Materials and methods

### Study subjects

The current cross-sectional analysis compared pQCT measures from three cohort studies. The Mobility and Independent Living in Elders Study (MILES) was established in 2012 to estimate the prevalence of age related diseases and risk factors for disability among Indians residing in rural south India [17]. A random sample of 562 men and women 60 years and over, were enrolled from Medchal Region of Telangana state of southern India. The response rate for men in MILES was 74%. Of the 495 men and women who underwent pQCT, all 245 men were included in this analysis.

The Osteoporotic Fractures in Men (MrOS) study is a prospective study designed to identify risk factors for fracture among older men (65 years and more); 5994 men were recruited in 2000–2002 at six different geographic regions of the US. Key recruitment methods included mailings using community and provider contact lists, regional and senior newspaper advertisements, and presentations targeted to seniors. Sites used a centrally developed recruitment brochure. Response to mass mailings at some sites surpassed 10–15% and appointment show rates averaged above 85%. The final number enrolled in MrOS was 5% more than the original recruitment goal of 5700 [18, 19]. The current analysis included men from the Minneapolis and Pittsburgh sites which obtained pQCT measures during the second visit of the study between 2005 and 2006. Of the 1180 participants who completed the second visit at these sites, 1148 (97%) US Caucasians were included in this analysis.

The Tobago Bone Health study was initiated as part of population based prostate cancer screening cohort study between 1997 and 2003 among men older than 40 years of age [20]. These men were recruited by word of mouth, poster, flyers, public health announcements, and health care workers and represent about 50% of the men age 40–79 residing on the island of Tobago [21]. Between 2004 and 2007, men in the cohort were invited to return for a repeat examination which included pQCT [22]. Of the 2153 men who underwent pQCT, 828 men with all 4 grandparents of African ancestry and aged 60 years and over were included in this analysis.

### pQCT and calibration

pQCT scans on the radius and tibia were performed using the Stratec XCT-2000 (Stratec Medizintechnik, Pforzheim, Germany) in MILES, Tobago, and the Pittsburgh site of MrOS study. The Minneapolis site of MrOS performed the scans using Stratec XCT-3000 scanner. Technicians followed a standardized protocol for positioning and scanning of each subject. First, a coronal scout view of a 40-mm section encompassing the distal end of the radius or tibia was obtained. Second, the flat portion of the radio-carpal joint or tibia endplate was marked in the scout view and the scanner gantry moved a fixed distance proximal and along the subject’s arm or leg from the marked position. The length of tibia was measured from the medial malleolus to the medial condyle of the tibia. Radius length was measured from olecranon to ulna styloid process. Scans were taken at 4 and 33% of the length of radius and at 4%, 33 and 66% of the length of tibia. Subject scans were repeated if artifacts due to motion or beam hardening were present. To monitor the stability of the pQCT scanners, a manufacturer supplied cylindrical quality assurance (QA) phantom was scanned daily before subject scans were acquired. The phantom is 5 cm in diameter with a hydroxy-apatite core manufactured to have a uniform absorption value comparable to trabecular bone of moderate density. The absorption and cross-sectional area measurements recorded for the phantom are automatically stored in a phantom QA log file generated by the scanner software. This QA log file was checked periodically to ensure that the scanner calibration did not drift. In addition, a European Forearm Phantom (EFP) was scanned at the beginning and end of each study to ensure that the scanner calibration did not drift from factory settings. The EFP has four distinct density zones that mimic trabecular and cortical bone typical for a distal and proximal radius. Three repeat scans were taken and analyzed for comparing the density. Fit coefficients were derived and correction factor for MILES scanner density measurement was applied (MILES = 1.02 (MrOS) + 1.9). The Tobago study scanner did not have any difference when compared with the MrOS scanner. All pQCT scans were analyzed by a single investigator using the manufacturer software package version 6.00 for the XCT scanners. This software provides a suite of segmentation options to quantify total, trabecular and cortical bone properties from each pQCT image. Before each image was analyzed it was checked for artifacts due to motion or beam hardening. Scans with artifacts were not analyzed. All 4% radius and tibia scans were analyzed using the CALCBD option with an automatic gradient search (contour mode 2) applied to segment bone from the soft tissue background and concentric peeling (peelmode 1, 45%) to segment trabecular and cortical bone. Proximal scans acquired at the 33 and 66% limb locations were segments using a fixed threshold of 710 mg/cm3(Cortmode1). Coefficients of variation (CVs) were determined for pQCT scans by replicating measurements on 15 subjects (CV ≤ 2.1%). Though there are differences in the XCT 2000 and 3000 scanners, these machines were calibrated at the factory to the European forearm phantom. Even with the slight differences in technical parameters such as effective X-ray beam energy position, the EFP calibration step ensured that volumetric density derived on each scanner are directly comparable. None of the scanners had a calibration drift due to service issues during the study.

### pQCT parameters

For this analysis, we focused on the following pQCT parameters that are physiologically important in skeletal aging: at the 4% site of radius and tibia—trabecular vBMD and Strength Strain Index (SSIp); at the 33% sites of radius and tibia—cortical vBMD, cortical thickness, endosteal circumference, and SSIp. vBMD was chosen as it is an indicator of bone matrix mineralization or mechanical quality of the solid bone tissue. Endosteal circumference and cortical thickness were chosen as they represent bone geometry and strength. SSIp was chosen as it predicts the failure load [23] and also has been shown to be a good predictor of long bone bending [24]. All these parameters also have age-related changes due to adaption of stress, strain, and load on the bone [25, 26].

### Other measures

Information on demographics, lifestyle factors, self-reported health status, and direct measures of body weight and height were obtained. Body mass index (BMI) was calculated as body weight in kilograms divided by height in meters squared. Fracture history, among the US Caucasians was based on self-report of fractures at baseline after age 50 years and incident fractures from the baseline visit to the second visit. Among rural South Indians, fracture history was based on the participants’ recall of a fracture in the last 5 years; and among the Afro-Caribbean men, this was based on health history of fracture event ever. Self-reported history of falls in the past 12 months was collected in all three studies. Information on diabetes was based on glucose levels ≥ 126 mg/dL (after a minimum of an 8-h fast), or self-report of diabetes or insulin or hypoglycemic medications among rural South Indians and Afro-Caribbean’s whereas among US Caucasians, it was self-reported. Hypertension among the Afro-Caribbean and rural South Indian populations was based on self-report, or medication inventory or blood pressure assessment. Hypertension among US Caucasians was self-reported. Grip strength was measured using hand-held dynamometers among all the three studies. Ever smoking status was based on self-reported current and past smoking status in all the three populations. Drinking alcohol among US Caucasians was having at least 12 drinks in the past 12 months. Among Afro-Caribbean population, it was based on the question of how many drinks in a typical week for the past 12 months. Among rural South Indian population, it was based on current consumption of alcohol.

### Statistical analysis

Characteristics of the three groups are expressed as percentages or mean ± standard deviation (SD), confidence intervals and were compared by ANOVA or chi square. Any pQCT parameter with a value of mean ± 3 SD was identified within each study and removed from analysis. pQCT parameters were compared across the three groups using general linear models (GLM). Comparisons were performed adjusting for age, height, weight, and grip strength. Percentage and standard deviation differences in the mean pQCT parameters were also performed keeping US Caucasians as the referent group. Standard deviation differences presented are the difference in mean pQCT parameters in terms of number of SDs based on the US Caucasians. Results were considered statistically significant when a *p* value was less than 0.001 with Bonferroni correction for multiple comparisons. Statistical analyses were performed using SAS (version 9.3; SAS Institute, Cary, NC, USA).

## Results

### Characteristics of the populations

When compared to US Caucasians (77.2 ± 5.2), rural South Indian (68.2 ± 6.6), and Afro-Caribbean (68.8 ± 6.8) men were significantly younger by more than 8 years (Table 1). Rural South Indian men had significantly lower body weight (55.9 ± 11.5), height (160.6 ± 5.6), and BMI (21.6 ± 3.9) compared to US Caucasians and Afro-Caribbean men. Rural South Indian men had significantly lower grip strength (20 ± 8.1 kg) compared to US Caucasians (37.8 ± 7.7 kg), and Afro-Caribbeans (35.9 ± 10.8 kg). Afro-Caribbeans had lower prevalence of smoking (past and current), whereas alcohol consumption was significantly higher in rural South Indian men compared to US Caucasians and Afro-Caribbeans. Rural South Indian men were less likely to report a past history of fall (9%) and fractures (4.9%) compared to US Caucasians and Afro-Caribbean men. The prevalence of hypertension among the older men was similar among rural South Indian and US Caucasians whereas Afro-Caribbean men had significantly higher rates of hypertension with two thirds being hypertensive. Afro-Caribbean men were significantly more likely to be diabetic (29.7%) compared to rural South Indians (18.8%) and US Caucasians (15.6%). Based on self-report of current health status, significantly fewer rural South Indian men opined their health status as good (46.1%) compared to 86% of US Caucasians and Afro-Caribbean.

**Table 1:** Characteristics of the study populations: Rural South Indian, US Caucasian, and Afro-Caribbean older men

Characteristics	US Caucasian (*N* = 1148)		Rural South Indian (*N* = 245)		Afro-Caribbean (*N* = 828)	
Age in years [mean ± SD (95% CI)]	77.2 ± 5.2	(76.9,77.6)	68.2 ± 6.6*	(67.5,68.9)	68.8 ± 6.8*	(68.4,69.3)
Weight (kg) [mean ± SD (95% CI)]	84.1 ± 13.4	(83.3,84.9)	55.9 ± 11.5*	(54.2,57.6)	81 ± 15.2 *	(80.2,82.1)
Height (cm) [mean ± SD (95% CI)]	173.1 ± 6.8	(172.7173.5)	160.6 ± 5.6*	(159.8161.5)	173.2 ± 9.2	(172.9173.8)
BMI (kg/m2) [mean ± SD (95% CI)]	28 ± 4	(27.7,28.2)	21.6 ± 3.9*	(21.1,22.1)	26.9 ± 4.7 *	(26.7,27.3)
Grip Strength (kg) [mean ± SD (95% CI)]	37.8 ± 7.7	(37.3,38.2)	20 ± 8.1*	(19.2,21.3)	35.9 ± 10.8 *	(36.2,37.4)
Ever smoked [% (95% CI)]	63.9	(61.2, 66.7)	75.1*	(69.7, 80.5)	31.9*	(28.7, 35.1)
Drinks Alcohol [% (95% CI)]	61.1	(58.3, 63.9)	71.4*	(65.8, 77.1)	50.7*	(47.4, 54.3)
History of fracture [% (95% CI)]	24.3	(21.8, 26.8)	4.9*	(2.2, 7.6)	14.8*	(12.4, 17.2)
History of fall [% (95% CI)]	25.7	(23.2, 28.2)	9*	(5.4, 12.6)	16*	(13.5, 18.5)
Hypertension [% (95% CI)]	52.7	(49.8, 55.6)	52.7	(46.4, 58.9)	66.9*	(63.7, 70.1)
Diabetes [% (95% CI)]	15.6	(13.5, 17.7)	18.8	(13.9, 23.7)	29.7*	(27.2, 33.4)
Health status opinion—Good/excellent [% (95% CI)]	86.2	(84.2, 88.1)	46.1*	(39,9, 52.4)	85.9	(84.3, 88.9)

Please see the “Materials and methods” section for ascertainment methods

Means expressed in the table are least squares means (LS-Means)

*p value < 0.05 when compared with the US Caucasian population

### vBMD, bone structure, and structural geometry

Rural South Indian men had significantly lower trabecular vBMD at the radius (− 28.4%) and tibia (− 24.8%) compared to US Caucasians (Table 2). Cortical thickness at the radius and tibia (− 13.4 and − 16.6%) were significantly lower when compared with US Caucasians. Endosteal circumference was significantly higher at the radius (9.4%) and tibia (12.1%) in rural South Indians compared to US Caucasian men. Cortical vBMD at the radius (− 0.7%) and tibia (− 0.1%) were similar to US Caucasians. Except for SSIp at 4% tibia (− 20.7%) which was significantly lower, SSIp at 4% radius, 33% radius and 33% tibia were similar to US Caucasians. These differences were independent of age, height, weight, and grip strength.

**Table 2:** Mean and Percent difference in the pQCT parameters of rural South Indian and Afro-Caribbean older men compared with US Caucasian (age, height, weight, and grip strength adjusted)

	US Caucasian	Rural South Indian		Afro-Caribbean men	
pQCT parameters	Mean (95% CI)	Mean (95% CI) (*p* value)	% difference^a^	Mean (95% CI) (p value)	% difference^a^
Trabecular vBMD—radius 4%	197.3	141.2	− 28.4	201.7	2.2
	(193.9, 200.7)	(132.8, 149.6)		(198.2, 205.2)	
		(< 0.0001)*		(0.308)	
Trabecular vBMD—tibia 4%	229.9	172.9	− 24.8	227.5	− 1.0
	(226.9, 232.8)	(165.6, 180.2)		(224.4, 230.5)	
		(< 0.0001)*		(0.9341)	
Cortical vBMD—radius 33%	1164.5	1156.5	− 0.7	1201.6	3.2
	(1162.3, 1166.8)	(1151,1162.1)		(1199.3, 1203.9)	
		(0.0591)		(< 0.0001)*	
Cortical vBMD—tibia 33%	1140.7	1139	− 0.1	1168.3	2.4
	(1138.5, 1142.9)	(1133.5, 1144.5)		(1165.9, 1170.6)	
		(1.00)		(< 0.0001)*	
Cortical thickness—radius 33%	3.29	2.85	− 13.4	3.49	6.1
	(3.26, 3.34)	(2.76, 2.94)		(3.46, 3.53)	
		(< 0.0001)*		(< 0.0001)*	
Cortical thickness—tibia 33%	5.59	4.66	− 16.6	5.36	− 4.1
	(5.53, 5.64)	(4.53, 4.79)		(5.30, 5.42)	
		(< 0.0001)*		(< 0.0001) *	
Endosteal circumference—radius 33%	21.2	23.2	9.4	21.7	2.4
	(21, 21.4)	(22.5, 24)		(21.4, 22)	
		(< 0.0001)*		(0.0531)	
Endosteal circumference—tibia 33%	39.7	44.5	12.1	46	15.9
	(39.2, 40.1)	(43.3, 45.6)		(45.5, 46.5)	
		(< 0.0001)*		(< 0.0001)*	
SSI p—radius 4%	490.3	459	− 6.4	575.1	17.3
	(481.3, 499.2)	(436.9, 481.1)		(565.9, 584.2)	
		(0.0657)		(< 0.0001)*	
SSI p—tibia 4%	2363.5	1874.7	− 20.7	2530.3	7.1
	(2318.3, 2408.7)	(1763.6, 1985.9)		(2483.3, 2577.4)	
		(< 0.0001)*		(< 0.0001)*	
SSI p—radius 33%	347.3	346.7	− 0.2	420.9	21.2
	(342.3, 352.3)	(334.5, 359)		(415.8, 426)	
		(1.00)		(< 0.0001)*	
SSI p—tibia 33%	1999	1883.5	− 5.8	2460.5	23.1
	(1974.9, 2023.1)	(1824, 1943)		(2435.3, 2485.6)	
		(0.0051)		(< 0.0001)*	

^a^Percent difference compared to US Caucasian

*Difference compared to US Caucasians is significant (*p* < 0.001)

Afro-Caribbean men had similar trabecular vBMD compared to US Caucasians. All the other assessed pQCT parameters were significantly higher among Afro-Caribbean men except tibial cortical thickness (− 4.1%), which was significantly lower and endosteal circumference of radius which was not different compared with US Caucasians (Table 2).

**Fig. 1:**
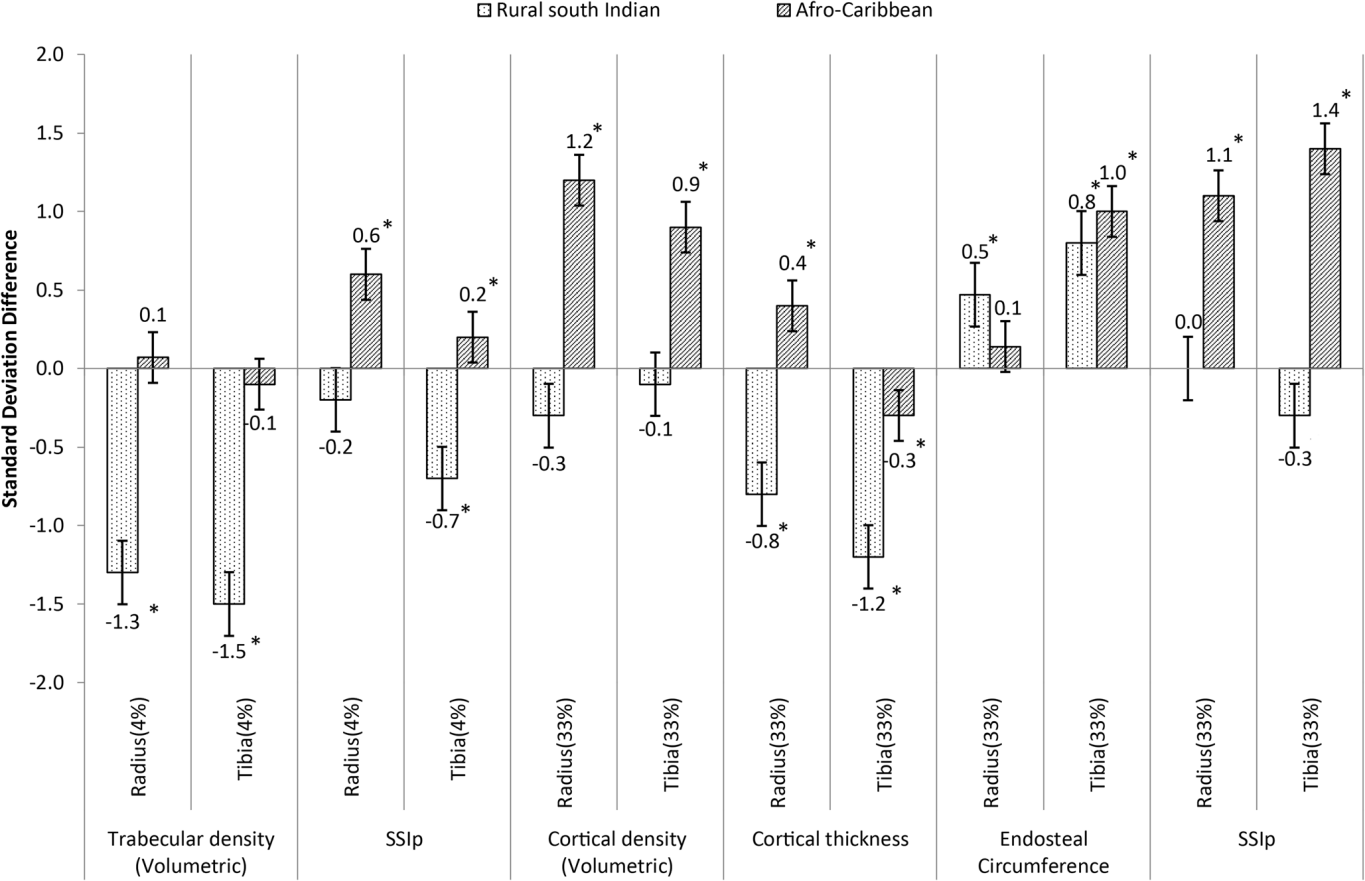
Standard deviation difference of mean pQCT parameters among rural South Indian and Afro-Caribbean compared to US Caucasian older men (age, height, weight, and grip strength adjusted).*Difference compared to US Caucasians significant (*p* < 0.001); Error bars represent 95% confidence intervals of the SD difference

Standard deviation differences in the skeletal parameters, independent of age, height, weight, and grip strength were compared to US Caucasians (Fig. 1). Rural South Indian men compared to US Caucasians had lower (− 1.3 to − 1.5 SD) trabecular vBMD, cortical thickness (− 0.8 to − 1.2 SD), and SSIp at 4% tibia (− 0.7 SD); and higher endosteal circumference (0.5 to 0.8 SD). Among the Afro-Caribbean men, cortical vBMD, tibial endosteal circumference, radial cortical thickness, and SSIp were 0.2 to 1.4 SD higher than US Caucasian men. Tibial cortical thickness was −0.3 SD lower compared to US Caucasians.

## Discussion

Rural South Indian men compared to US Caucasians had lower trabecular vBMD, cortical thickness, and SSIp at 4% tibia; higher endosteal circumference; but similar cortical vBMD, radial SSIp, and SSIp a 33% tibia. The European Male Aging Study (EMAS) which compared south Asians and whites living in Manchester, UK had comparable observations of lower tibial trabecular vBMD, tibial, and radial cortical thickness; similar radial cortical vBMD; but on the contrary, radial trabecular vBMD was similar and tibial cortical vBMD was lower [27]. In the current study, Afro-Caribbean men compared with US Caucasians had higher cortical vBMD, tibial endosteal circumference, SSIp, and radial cortical thickness; lower cortical thickness at the tibia; and similar trabecular vBMD. These differences suggest geographic/racial differences in measures of bone structure, geometry, and strength.

With increasing age, cortices get thinner, the cortical envelope becomes more porous, and trabecular bone mass declines, all of which contribute to increased bone fragility among older adults [25, 26, 28, 29]. The rural South Indian older men in our study had lower trabecular vBMD and cortical thickness in both the upper and lower limbs. This in combination with lower 4% tibial SSIp suggests increased susceptibility to fractures and future morbidity among rural South Indian men. Cortical vBMD is a determinant of intrinsic stiffness of bone tissue. The rate of loss of cortical vBMD is steady or slower from 50 years till 75 years of age, compared to trabecular vBMD [30, 31]. Though the US Caucasians were older, rural South Indian men had similar cortical vBMD and significantly lower trabecular vBMD.

Our results suggest that the patterns of pQCT parameter differences across different geographic/race groups were similar to the DXA-based aBMD comparisons published earlier [15, 32]. The aBMD at the femoral neck, total hip, and lumbar spine were significantly higher among Afro-Caribbean compared to US Caucasian men. The aBMD of US Asians (significant for total hip), Hong Kong Chinese (significant for femoral neck, total hip, and lumbar spine), and South Koreans (significant for total hip and lumbar spine) was lower compared to US Caucasians [15]. To our knowledge, there is lack of data on aBMD comparisons among older Indian men. Healthy Indian men aged 20–29 had significantly lower aBMD at the hip, forearm, and lumbar spine when compared to the third US National Health and Nutrition Examination Survey (NHANES III, 1988–1994) [33]. In contrast, a study of UK white and Indian men aged 20–40 years showed similar aBMD [34]. These observations suggest conflicting directionality of aBMD and pQCT differences among different racial/geographic groups. Among the US MrOS cohort, several pQCT parameters were strongly associated with non-vertebral fractures among older men and these associations were independent of aBMD [35]. Considering this, it is important to focus on vBMD, bone structure, and structural geometry in furthering our understanding of geographical differences in hip fractures.

Our rural South Indian population compared to US Caucasians had lower trabecular vBMD, lower cortical thickness, lower 4% tibial SSIp, and higher endosteal circumference suggesting an increased fragility of radius and tibial bones among the Indian population. Conversely, self-reported history of fracture was lower among the rural South Indian men. This may reflect recall bias and needs to be interpreted with caution. In addition, the time frame for fracture history recall was shorter among rural South Indian men (past 5 years), than the US Caucasian men (fracture since age 50) or Afro-Caribbean men (ever had fracture). However, age standardized incidence of hip fractures for Indian men is around 122 per 100,000 and for US men is 155 per 100,000 [11], which suggests a lower fracture burden among Indian men. These rates were based on a single study in one district (Rohtak) of northern India in 2009 from four orthopedic hospitals and hence, may not be representative of hip fracture rates across the diverse country of India [36]. National population-based data are needed for rates of hip fractures in India.

Our results suggest that the rate of hip fracture at least among rural Indians should be higher than US white men. The lower fracture prevalence among rural South Indian men is contrary to our expectations but could reflect the following. First, the fractures were self-reported and not adjudicated. Second, Indians have lower life expectancy at birth (68 years) compared to US (79 years) and Trinidad and Tobago (70 years) [37]. In 2012, life expectancy at age 60 years for Indian and Trinidad and Tobago men was 16 years compared to 21 years for US men [38]. This lower life expectancy at birth and at age 60 may lead to fewer fractures due to competing mortality. Third, Indians have a lower hip axis length [39] which could impact fracture risk.

The Afro-Caribbean population had significantly higher cortical vBMD, radial cortical thickness, endosteal circumference, SSIp, and similar trabecular vBMD compared with US Caucasians. This suggests that the skeleton of this population is less fragile in comparison to US Caucasians. These observations are consistent with the lower fracture rates among men of Afro-Caribbean ancestry [32].

It has been estimated that around 50 million people in India are osteoporotic [40] and this is likely due to combination of multiple factors including genetic, nutritional, lifestyle, and smaller skeletal size [41]. The increasing aging population in India coupled with increased osteoporosis will impact the number of fractures observed among Indian elderly. The current analysis adds to the literature and presents a scenario of fragile bones among rural South Indian older men.

There are several potential limitations to the current analysis. We used a cross-sectional design and hence cannot infer causation. The sample size of the Indian population was lower and was restricted to one specific rural southern region in India; thus generalization of these finding to the larger Indian population needs to be done with caution. The geographical differences between the populations may also reflect other factors including genetic, lifestyle, chronic diseases, concurrent medications, and physical activity. Different ascertainment methods of the covariates limited harmonization of the data and we did not include them in the models. In this analysis, we adjusted the covariates age, height, weight, and grip strength, which were collected similarly in all the three studies. Another possible limitation was the partial volume effect, which may underestimate cortical vBMD due to thinner cortices. However, the current analysis also has several notable strengths. First, to our knowledge, this is the first description of pQCT parameters among older men from rural south India and the first comparison to a large well-characterized population of older US Caucasian men and Afro-Caribbean men. Second, we performed cross calibration of each pQCT machine using a European forearm phantom.

In conclusion, compared to US Caucasians, rural South Indian men had reduced bone strength primarily because of lower trabecular vBMD and Afro-Caribbean men had greater bone strength primarily because of higher cortical vBMD. These findings suggest an underlying higher risk of osteoporotic fractures among rural Indian men that may translate to a greater future fracture burden. Though there have been estimations of hip fracture rates among Indians [40, 42], the relationship between the pQCT measures studied and fracture risk has not been established in India.
